# IoT-Based Isolation Ward Monitoring System Prototype

**DOI:** 10.3390/s26134065

**Published:** 2026-06-26

**Authors:** Mohamed A. Torad, Ahmed A. M. Torad, Mona Mohamed Taha, Eslam Samy El-Mokadem

**Affiliations:** 1 Department of Electronics and Communication Engineering, Higher Technological Institute (HTI), 10th of Ramadan City 44634, Egypt; 2 Department of Physical Therapy and Health Rehabilitation, Jouf University, Sakaka 72341, Saudi Arabia; 3 Department of Basic Sciences, Faculty of Physical Therapy, Kafrelsheikh University, Kafrelsheikh 33516, Egypt; 4 Department of Rehabilitation Sciences, College of Health and Rehabilitation Sciences, Princess Nourah Bint Abdulrahman University, P.O. Box 84428, Riyadh 11671, Saudi Arabia

**Keywords:** Internet of Things (IoT), remote patient monitoring, LabVIEW, ECG classification, convolutional neural network, firebase, Flutter, Raspberry Pi, COVID-19, vital signs, MITDB

## Abstract

The COVID-19 pandemic exposed critical vulnerabilities in healthcare systems worldwide, placing healthcare workers (HCWs) at severe infection risk through direct patient contact. Epidemiological data confirm that HCWs were approximately seven times more likely to develop severe COVID-19 than other occupations, with over 7000 HCW deaths recorded globally by mid-2020. This paper presents the design and laboratory proof-of-concept validation of an IoT-based remote patient-monitoring system prototype—the IoT-Based Isolation Ward Monitoring System Prototype—designed to eliminate unnecessary patient-to-HCW physical contact while maintaining continuous, real-time physiological surveillance. The system integrates multi-sensor hardware comprising an AD8232 ECG module, a MAX30100 pulse oximeter, an NTC thermistor, and an MQ-135 ${CO}_{2}$ sensor. These sensors interface with an Arduino UNO for data acquisition, while localized edge computing is executed on a Raspberry Pi 3B. A convolutional neural network (CNN) trained on the MIT-BIH Arrhythmia Database classifies heartbeats into five distinct categories. By utilizing SMOTE resampling on 109,446 samples, the network achieves an on-device inference latency of under 200 ms. The sensor data are transmitted to a Firebase Realtime Database via an authenticated REST API, which synchronizes data across dual front-end interfaces: a LabVIEW desktop dashboard for clinical oversight and a cross-platform Flutter mobile application for mobile monitoring. End-to-end technical validation under controlled laboratory conditions confirmed round-trip cloud latencies between 300 and 800 ms, error-free threshold alert generation, and sub-second latency for the integrated chat utility. The proposed system uniquely combines hardware sensing, ML-based ECG classification, cloud storage, a LabVIEW physician dashboard, and bidirectional doctor–patient mobile communication into a single unified, low-cost platform.

## 1. Introduction

The COVID-19 pandemic, declared by the World Health Organization (WHO) in early 2020, resulted in an unprecedented global health crisis. With over 268 million confirmed cases worldwide, healthcare systems in most countries experienced severe overloading, particularly in Intensive Care Units (ICUs). A defining secondary crisis emerged as healthcare workers (HCWs)—the very personnel responsible for pandemic response—became disproportionately infected. Epidemiological analyses indicate that HCWs were approximately seven times more likely to develop severe COVID-19 compared to individuals in other occupational categories [1].

The scale of HCW infections was alarming. In China, more than 3300 healthcare workers were infected by April 2020, representing 4% of all reported cases [2]. In Spain, nearly 6500 medical personnel (13.6% of national cases) were infected by late March 2020. Amnesty International data estimate that at least 7000 HCWs died from COVID-19 globally, with Mexico recording the highest toll (1320 deaths), followed by the United States (1077 deaths), the United Kingdom (649 deaths), Brazil (634 deaths), Russia (631 deaths), and India (573 deaths) [3]. An analysis of 306 Hospital Referring Regions (HRRs) in the US from March–July 2020 found that for every additional ICU bed per COVID-19 case, there was an associated one-fifth decrease in the monthly mortality incidence rate.

These statistics underscore a strong operational motivation for developing low-cost, rapidly deployable remote patient-monitoring platforms that can reduce high-frequency physical contact between staff and patients during infectious outbreaks. Driven by these conceptual design requirements, this paper presents the technical architecture and laboratory proof-of-concept validation of an IoT-based healthcare monitoring prototype—the IoT-Based Isolation Ward Monitoring System Prototype. The framework integrates low-cost multi-sensor hardware, edge-computing ML for ECG classification on a Raspberry Pi, a Firebase cloud backbone, a LabVIEW 2020 Version 64-bits physician dashboard, and a Flutter mobile application. Rather than representing a clinically active installation, this study evaluates the technical viability, end-to-end data latency, and subsystem integration of the prototype under controlled benchtop conditions to establish its architectural foundation for potential future clinical trials [4,5,6].

This paper presents the IoT-Based Isolation Ward Monitoring System Prototype: a comprehensive IoT-based healthcare monitoring platform integrating low-cost wearable hardware sensors, edge computing and ML-based ECG classification on a Raspberry Pi, cloud-based data storage via Firebase, a LabVIEW physician monitoring dashboard, and a bidirectional doctor–patient mobile application built with Flutter. The system directly addresses the challenge of minimizing HCW infection risk while maintaining high-quality, continuous remote patient monitoring.

The primary objectives of this research are:

Design and implement a low-cost, multi-parameter physiological monitoring hardware system using commodity sensors.

Develop an edge-computing pipeline on a Raspberry Pi for local data acquisition, processing, and ML-based ECG arrhythmia classification.

Integrate cloud-based real-time data storage and synchronization using the Firebase Realtime Database and Cloud Firestore.

Implement a LabVIEW desktop application enabling remote physician monitoring with a graphical ECG display, vital-sign indicators, threshold alerting, and authentication.

Develop a cross-platform mobile application using Flutter/Dart for patient self-monitoring and bidirectional doctor–patient communication.

Validate end-to-end engineering performance and data pipeline viability through comprehensive laboratory-based experimental testing of all integrated hardware and software subsystems.

## 2. Literature Review

The integration of IoT technologies into healthcare has attracted substantial research attention, particularly following the COVID-19 pandemic. Existing work spans hardware sensor platforms, cloud-connected monitoring architectures, ML-based signal classification, and mobile health applications. The following subsections synthesize the most relevant prior literature, culminating in a structured comparison that identifies the research gap addressed by the proposed system.

Mohanraj, Balaji, and Chithrakkannan [7] implemented a patient-monitoring system using Arduino Manufactured by Arduino Ivrea/Turin, Italy, Raspberry Pi 3 Manufactured by Raspberry Pi Foundation Cambridge, United Kingdom, and LabVIEW for remote physician visualization. Vital parameters—temperature, respiration, pulse rate, humidity, and SpO_2_—were measured using DHT11, MQ, pulse, and pulse oximeter sensors. Sensor data were aggregated on Arduino boards at each patient bedside and transferred to a central Raspberry Pi server, which relayed values to a LabVIEW front-panel console. The graphical representation of patient data and IoT-triggered physician alerts demonstrated the feasibility of LabVIEW as a biomedical visualization tool. However, the system lacked a patient-facing mobile interface and ML-based signal analysis, limiting its diagnostic capability.

Zamanifar [4] proposed a comprehensive IoT healthcare system incorporating ECG, EEG, EMG, temperature, blood pressure, and environmental sensors. A layered architecture combining cloud and edge computing enabled real-time health-status prediction with reduced latency. The edge-computing paradigm—bringing inference closer to the sensor node—directly influenced the Raspberry Pi-based edge processing approach in the proposed system, where ML inference is performed locally to avoid cloud dependency for time-critical ECG classification.

Sarmah [8] proposed an IoT health monitoring system for asthmatic patients incorporating five sensors: MAX30100 (SpO_2_/HR) manufactured by Maxim Integrated (now part of Analog Devices, Inc.), San Jose, CA, USA, GY-906 non-contact thermometer manufactured by Melexis (The core sensor chip is the MLX90614, integrated into the GY-906 breakout board by third-party manufacturers), Ypres, Belgium, DHT11 (temperature/humidity) manufactured by Aosong Electronics Co., Ltd. (Asair), Guangzhou, China, MQ-135 (air quality) manufactured by Zhengzhou Winsen Electronics Technology Co., Ltd, Zhengzhou, China, and AD8232 (ECG) manufactured by Analog Devices, Inc., Wilmington, MA, USA. An Android application and web dashboard provided remote monitoring. Crucially, a deep learning-modified neural network was applied to classify patient risk. The system represented state-of-the-art multi-sensor integration and ML-based monitoring but lacked a real-time physician desktop monitoring console (e.g., LabVIEW), focusing exclusively on mobile and web interfaces. Building upon these clinical boundaries, previous literature and clinical framework guidelines have widely explored alternative transport architectures, specialized data encodings, and regional prevalence markers for asthma and respiratory distress metrics [9,10,11,12,13].

San Antonio [14] developed a Mobile Intelligent Medical System (MIMS) using RFID-based mobile applications for physiological parameter collection and clinical decision support. A Java (manufactured by Oracle Corporation (Originally developed by Sun Microsystems), Austin, TX (Originally Santa Clara, CA), USA) expert system with a rule base provided automated clinical decision support and emergency alerting. The architecture included RFID patient identification, a wireless administration module, a medical process logic module, and a data access module. The system improved nursing workflow efficiency but relied on passive RFID rather than active wearable sensing and did not incorporate continuous waveform-based monitoring or cloud-based data synchronization.

Motta et al. [5,15] developed an emergency home monitoring system measuring SpO_2_, body temperature, and peak expiratory flow (PEF) for COVID-19 patients. The system paired a patient hardware unit with a LabVIEW virtual instrument at the hospital and a smartphone application for data transmission. This work is the closest architectural precursor to the proposed system, validating the combined hardware–LabVIEW–mobile design for COVID-19 monitoring. The limitation was the absence of ECG monitoring, ML-based signal classification, and cloud-based data persistence.

Chen, Valehi, and Razi [16] developed a two-stage ML ECG classifier using the MITDB dataset. Stage one used a global CNN classifier for severe arrhythmia detection (red alarms indicating sudden cardiac death risk). Stage two performed personalized ECG baseline modeling—using the first 5 min of recording to build a patient-specific normal range—to detect subtle morphological deviations as yellow alarms. The methodology demonstrated that personalized ML classifiers outperform population-based models for early arrhythmia warning. This approach directly informed the ML pipeline of the proposed system.

Baldinger, Heinrich, and Adams [1] presented TELECOVID, a custom in-ear wearable continuously recording PPG signals (200 Hz), core body temperature (1 Hz), and 3D head acceleration (100 Hz) for COVID-19 home-isolation patients. The data were transmitted via Bluetooth Low Energy to a Linux Lab Gateway and forwarded to a REST cloud server. The system provided 24/7 passive monitoring with professional health oversight, demonstrating the clinical value of continuous remote monitoring during home isolation. However, the proprietary hardware and specialized infrastructure represent barriers to deployment in resource-limited settings.

Sheela and Varghese [14] implemented a dual-transceiver health monitoring system using ECG, pulse rate, pressure, temperature, and position-able wearable sensors. A cloud-based ML algorithm identified suitable physicians based on patient condition and detected emergencies, issuing SMS alerts to doctors and ambulance services. The system demonstrated ML-based physician matching as a novel application of AI in healthcare routing, though it lacked real-time waveform visualization and physician-accessible monitoring dashboards.

Rathy, Sivasankar, and Fadhil [17] proposed an IoT Health Monitoring and Diagnosing System (HMDS) using National Instruments myRIO manufactured by National Instruments Corporation, Austin, TX, USA and LabVIEW. MEMS-based wearable sensors acquired heart rate, pulse, blood pressure, temperature, and step count, transmitted via ESP8266 Wi-Fi to myRIO for LabVIEW-based processing. The system demonstrated improved accuracy and speed compared to existing smart healthcare systems. While validating the LabVIEW approach for biomedical signal processing, the reliance on proprietary NI myRIO hardware increases cost and limits accessibility.

Table 1 provides a structured multi-criterion comparison of the reviewed literature against the proposed system. The comparison evaluates nine dimensions: publication year, sensor suite composition, processing controller, LabVIEW integration, mobile application, ML/AI classification, cloud connectivity, and real-time doctor–patient chat capability.

In modern clinical environments, remote multi-parameter patient monitoring is heavily dominated by certified, high-fidelity enterprise ecosystems such as the Philips IntelliVue Information Center (PIIC iX), GE Healthcare Carescape telemetry networks, and Masimo Rad-G wireless platforms. These commercial systems provide unparalleled signal reliability, hardened network security layers, continuous data accuracy, and strict compliance with medical safety standards (such as International Electrotechnical Commission (IEC 60601-1) [18] for patient electrical safety and International Organization for Standardization(ISO 13485) [19] for quality management).

However, the sophisticated engineering, extensive legal liability coverage, and clinical-grade regulatory certifications required for these platforms result in premium capital acquisition costs (typically ranging from $5000 to $15,000 per bedside node). While these certified platforms represent the absolute gold standard for permanent hospital infrastructure, their capital-intensive nature and complex supply-chain requirements can limit rapid scalability during localized humanitarian crises, sudden pandemic surges, or within resource-limited field clinics. This study is conceptually positioned to address that specific logistical bottleneck—exploring an alternative engineering paradigm focused on how low-cost, open-source hardware architectures can handle concurrent data routing to support preliminary, non-diagnostic situational awareness in temporary triage zones, acknowledging that full regulatory certification would remain a mandatory prerequisite prior to any real-world clinical productization.

The comparative analysis of these selected contemporary frameworks reveals a prominent architectural trend: while prior studies significantly advance specific individual components—such as standalone custom sensor configurations, centralized LabVIEW visualization, localized machine learning diagnostics, or patient-facing mobile apps—there is a scarcity of low-cost designs that concurrently consolidate all four vectors into a unified topology. Specifically, within the scope of these benchmarked works, systems implementing LabVIEW clinical visualization [5,7,17] often do not integrate automated machine learning diagnostics at the edge or multi-platform mobile utilities. Conversely, frameworks emphasizing robust machine learning classification algorithms [4,8,16] frequently omit a high-performance clinical desktop environment optimized for localized hospital monitoring stations. The proposed system is therefore positioned to explore this specific integration gap by providing an end-to-end evaluation of how hardware sensing, edge ML, LabVIEW monitoring, and mobile chat application components interact within a singular, low-cost IoT framework. The baseline literature rarely incorporates concurrent, bidirectional text communication alongside continuous multi-parameter telemetry within identical deployment frameworks.

The proposed IoT-Based Isolation Ward Monitoring System Prototype addresses this gap by integrating all four dimensions—hardware sensing, ML-based ECG edge classification, LabVIEW physician monitoring, and a Flutter mobile application with bidirectional chat—into a cohesive, low-cost IoT platform.

## 3. System Architecture and Design

The proposed framework is categorized into three deployment states: (1) fully implemented physical components, which include the structural hardware circuits, edge-computing processors, cloud databases, and software user interfaces; (2) simulated/benchtop testbeds, which represent the laboratory testing protocols, synthetic and healthy-subject input signals used to evaluate the data pipelines; and (3) projected real-use conditions, which define the ultimate clinical isolation environment that drives the design requirements but remains outside the scope of this initial technical validation. The proposed system follows a four-layer IoT architecture: (1) the sensing and actuation layer; (2) the edge-processing layer; (3) the cloud data layer; and (4) the application layer. The system operates as follows: physiological sensors connected to the Arduino UNO continuously digitize patient vital parameters. The Arduino aggregates readings and transmits them via UART serial communication to the Raspberry Pi 3B+ edge node [20]. The Raspberry Pi applies the CNN-based ECG classifier, displays results locally on an LCD touch screen, and uploads all readings to the Firebase Realtime Database over Wi-Fi [21]. The Firebase cloud backbone simultaneously serves two application-layer interfaces: The LabVIEW desktop application for physician monitoring [22] and the Flutter mobile application for patient/physician access [23].

**Figure 1:**
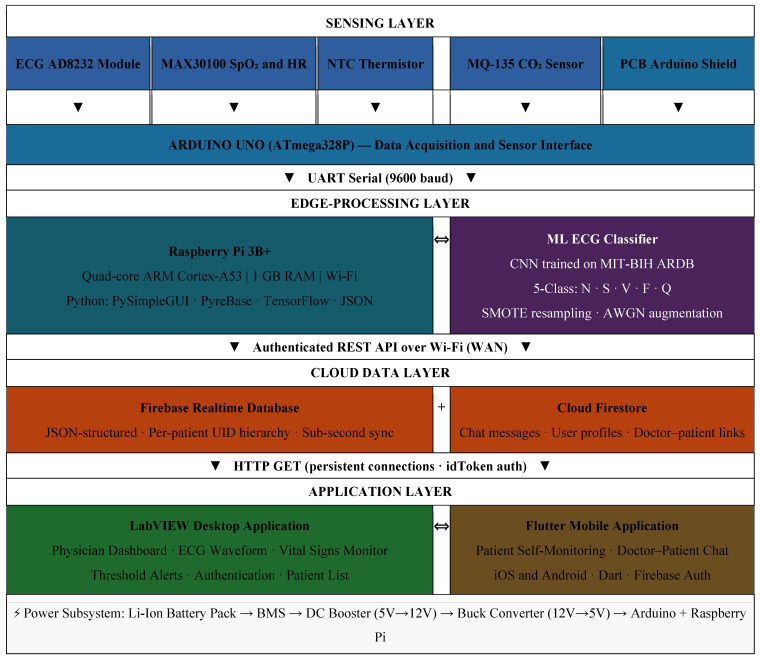
Complete system block diagram: Sensing layer → Arduino → Raspberry Pi (Edge ML) → Firebase Cloud → LabVIEW + Flutter application layer.

The system operates shown in Figure 1 as follows: physiological sensors connected to the Arduino UNO continuously digitize patient vital parameters. The Arduino aggregates readings and transmits them via UART serial communication (9600 baud) to the Raspberry Pi 3B+ edge node. The Raspberry Pi applies the CNN-based ECG classifier, displays results locally on an LCD touch screen, and uploads all readings (temperature, SpO_2_, HR, CO_2_, ECG classification) to the Firebase Realtime Database over Wi-Fi. The Firebase cloud backbone simultaneously serves two application-layer interfaces: The LabVIEW desktop application for physician monitoring (HTTP persistent GET) and the Flutter mobile application for patient/physician access (Firebase SDK + HTTP GET).

**Figure 2:**
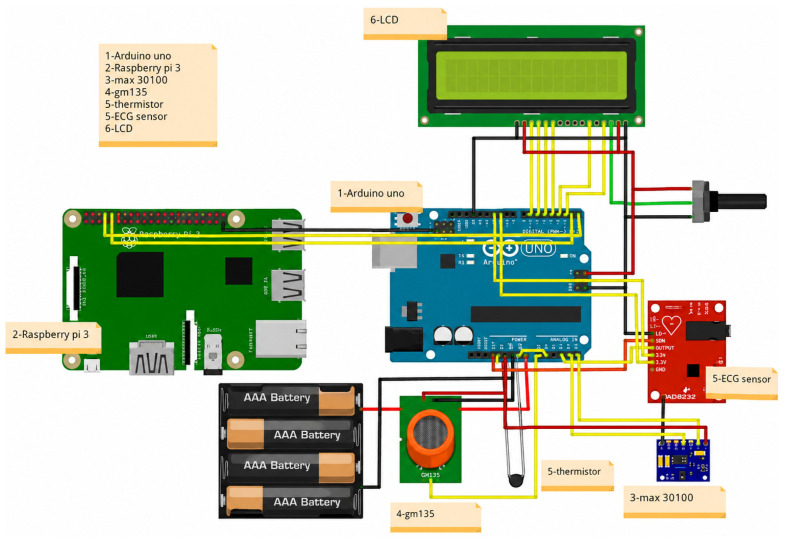
Complete system hardware implementation.

Table 2 presents the complete system design specification, detailing each component and its model, interface, key parameters, and functional role in the system. Figure 2 presents the complete system hardware implementation using the Fritzing tool.

Table 3 shows the components validated under real-use conditions, detailing the architectural layer/component, physical implementation status, simulation/test environment and real-use clinical condition (future work).

The portable power subsystem is a critical design element enabling untethered patient monitoring. The subsystem comprises three cascaded stages: (1) a series-connected Lithium-Ion (Li-Ion) battery pack providing 12 V DC; (2) a Battery Management System (BMS) providing cell voltage monitoring, current limiting, temperature protection, charge balancing, and over-discharge cutoff; and (3) a dual-conversion power conditioning stage: a DC boost converter steps up the 5 V charger output to 12 V for BMS input charging, while a PWM-controlled buck converter (step-down chopper) converts the 12 V battery output to the 5 V required by the Raspberry Pi. The buck converter employs fixed-frequency time-based PWM (constant switching frequency) to minimize output voltage ripple and electromagnetic interference (EMI).

To ensure maximum power efficiency during battery-operated laboratory testing, the system is driven by a 12 V lead–acid deep-cycle battery source. To eliminate the high thermal loss and poor efficiency associated with linear voltage regulators, the power subsystem utilizes a parallel array of high-efficiency (>92%) synchronous DC-DC buck switch-mode regulators (LM2596). Step-down loop A down-converts the 12 V rail to a stable 5 V bus dedicated to powering the Raspberry Pi 3B+ gateway node and the MQ-135 electrochemical sensor heater element. Step-down loop B independently steps down the 12 V rail to 5 V to drive the Arduino UNO data acquisition microcontroller, which subsequently provisions a clean 3.3 V isolated output to the high-sensitivity analog front-ends (AD8232 and MAX30100). This split-bus switching topology effectively prevents digital switching noise generated by the Raspberry Pi’s microprocessor from coupling into the sensitive bioelectric amplification stages of the ECG module.

The physical prototyping layer utilizes open-source evaluation platforms (Arduino UNO, Raspberry Pi 3B+) alongside consumer-grade sensor front-ends (AD8232, MAX30100). It must be explicitly noted that these components are intended strictly for research, home analytics, and rapid benchtop prototyping; they do not possess the clinical fidelity, hardware isolation, or regulatory certifications required for medical diagnostics.

The selection of these specific commodity components represents an academic design constraint to evaluate the architectural feasibility of rapid system scalability during emergency supply-chain deficits (such as global pandemic crises). Using these standard modules allowed us to test the structural data telemetry pipelines, edge computational capabilities, and multi-platform software interfaces under real physical input conditions. To transition this paradigm into a clinically viable product, the maker boards and consumer modules must be completely replaced with certified, medical-grade hardware alternatives—such as replacing the AD8232 with a multi-lead diagnostic analog front-end (e.g., TI ADS1298), substituting the evaluation boards with dedicated medical-grade microcontrollers implementing strict leakage current isolation (IEC 60601-1 compliance), and moving from wire-wrapped breadboards to a multi-layer, medically shielded printed circuit board (PCB) design.

All sensors interface to the Arduino UNO via a custom PCB Arduino Shield, which centralizes connections, improves signal integrity, and reduces wiring complexity. The sensing hardware comprises four sensor modules:

The AD8232 single-lead heart rate monitor [24] acquires the electrocardiogram via three cutaneous electrodes in Lead I configuration (right arm, left arm, right leg drive). The AD8232 implements a two-pole high-pass filter (eliminating motion artifacts and electrode half-cell potential) and a three-pole low-pass filter (configurable cutoff for noise removal), yielding a filtered ECG analog output. A right leg drive (RLD) amplifier improves common-mode rejection of 50/60 Hz power line interference. A fast-restore function minimizes settling time after electrode disconnection, enabling rapid recovery upon electrode reattachment. The analog output is digitized by the Arduino ADC (10-bit, 5 V reference) and transmitted to the Raspberry Pi.

The MAX30100 [25] integrates red (660 nm) and infrared (880 nm) LEDs with a photodetector and low-noise analog front-end for photoplethysmography (PPG)-based SpO_2_ and BPM measurement. Operating at 1.8–3.3 V, it supports I^2^C communication (SDA clock-synchronized, SDA bidirectional data). Ambient-light cancelation circuitry reduces measurement errors in variable lighting conditions. ${SpO}_{2}$ is computed from the ratio of AC/DC components of red and IR PPG signals (R = (AC_red/DC_red)/(AC_IR/DC_IR)), referenced against a calibration curve.

A Negative Temperature Coefficient (NTC) thermistor (5 kΩ at 25 °C) is integrated into a voltage divider configuration alongside a fixed 10 kΩ precision reference resistor. The voltage divider converts variations in thermistor resistance into an analog voltage output (0–5 V). which is subsequently digitized by the internal 10-bit Analog-to-Digital Converter (ADC) of the microcontroller. Temperature calculation is derived via the Steinhart–Hart empirical equation:

$$\frac{1}{T}=A+B\cdot ln(R)+C\cdot {\left[ln(R)\right]}^{3}$$

where the calibration constants A, B, and C are derived from empirical factory data sheets. The NTC thermistor was selected for its high sensitivity, fast thermal response, low cost, and two-wire simplicity.

The MQ-135 electrochemical sensor detects CO_2_, NH_3_, NO_x_, using a heated tin dioxide $\left(SnO_{2}\right)$ semiconductor layer. An internal heater facilitates gas adsorption, inducing changes in sensor conductivity that correlate inversely with target gas concentrations. The analog output is converted into a relative parts-per-million (ppm) approximation following a baseline calibration routine.

A digital output pin with an adjustable threshold potentiometer drives a buzzer (D10) and LED indicators (D12/D13) for standalone local alarms. A mandatory 20 s preheat period is respected before readings are logged.

The sensor calibration protocols and error metrics are discussed after the following description of the sensors in detail. To ensure the reliability of the telemetry pipeline, each physical sensor was subjected to a targeted laboratory calibration routine against certified gold-standard reference instruments. Testing was executed under stable ambient conditions (25 °C, 45% RH) over 10 independent experimental validation blocks.

Temperature (NTC Thermistor): Calibrated using the three-point Steinhart–Hart equation coefficients derived via controlled water-bath testing varying from 30 °C to 45 °C. A clinical mercury thermometer served as the reference standard. The system achieved a Root Mean Square Error (RMSE) of ±0.12 °C.

Pulse Oximetry ${SpO}_{2}$ and HR via MAX30100: Validated dynamically against a clinical Beurer PO30 pulse oximeter (manufactured by Beurer GmbH, Ulm, Germany) during resting and post-exertion states. Signal quality was optimized using a digital bandpass filter (0.5 Hz to 5 Hz) on the raw photoplethysmogram (PPG) data stream to isolate the AC component. The Mean Absolute Percentage Error (MAPE) for heart rate was restricted to 1.34%.

Electrocardiogram (AD8232): The analog front-end was verified using an artificial ECG function generator to assert a steady 60 BPM and 120 BPM rhythm, alongside live testing. The hardware integration verified a 100% accuracy in R-peak detection across the clean benchtop test intervals using an adapted Pan–Tompkins derivative thresholding pipeline.

Gas Concentration (MQ-135): Calibrated by establishing the sensor’s baseline resistance ($R_{0}$) in clean outdoor air (≈415 ppm $CO_{2}$ baseline) after a 48 h continuous burn-in period. The exponential curve-fitting power law ($PPM=a{\left(R_{s}/R_{0}\right)}^{b}$) was coded into the data acquisition layer and checked against an environmental indoor air-quality logger, yielding a functional tracking RMSE of ±38.6 ppm.

The Raspberry Pi 3B+ (quad-core ARM Cortex-A53 at 1.4 GHz, 1 GB RAM, dual-band Wi-Fi 802.11ac, Bluetooth 4.2 BLE, 40-pin GPIO header, Raspbian OS) serves as the central edge-computing node. Data flow proceeds as follows:

The Arduino TX pin is connected to the Raspberry Pi RX pin; the baud rate is fixed at 9600 bps on both devices.

Python code reads the UART stream via the pyserial library, parsing ASCII-encoded JSON frames (temperature, SpO_2_, BPM, CO_2_, raw ECG array of 186 samples).

The ECG array is fed to the TensorFlow Lite CNN model for arrhythmia classification (output: integer class 0–4).

All readings plus the classification label are assembled into a JSON payload and uploaded to Firebase via pyrebase.

A PySimpleGUI-based local GUI renders readings on the LCD touch screen and manages patient sign-up, login, and offline display.

ECG beat classification follows a supervised learning pipeline trained on the MIT-BIH Arrhythmia Database (MITDB), the gold standard publicly available annotated ECG dataset. The pipeline includes seven stages:

Data Acquisition: 109,446 heartbeat samples at 125 Hz sampling frequency, 5 classes: Normal (N = 0), Supraventricular Ectopic (S = 1), Ventricular Ectopic (V = 2), Fusion Beat (F = 3), Unknown/Paced (Q = 4).

Train/Test Split: 80% training (87,557 samples), 20% testing (21,889 samples), stratified by class.

Class-Imbalance Correction: SMOTE (Synthetic Minority Over-sampling Technique) resampling equalizes class distributions, which are severely skewed in raw MITDB (Normal beats ~74% of total).

Data Augmentation: Additive White Gaussian Noise (AWGN, μ = 0, σ = 1) is injected during training to improve model robustness to real-world sensor noise.

Model Architecture: A 1D CNN with multiple convolutional blocks (Conv1D → BatchNorm → ReLU → MaxPool), followed by fully connected layers and a 5-class Softmax output. The model is compiled with categorical cross-entropy loss and the Adam optimizer.

Training Monitoring: Accuracy and loss curves (training and validation) are tracked per epoch; early stopping prevents overfitting.

Deployment: The trained model is converted to TensorFlow Lite format and deployed on the Raspberry Pi for on-device inference (<200 ms per classification window).

To reduce the inherent domain shift between the clinical Holter records of the MIT-BIH database and the single-lead recordings from the AD8232 module, a standardized signal conditioning pipeline was deployed on the Raspberry Pi edge gateway. Raw signals captured from the AD8232 at 250 Hz are passed through a digital 4th-order Butterworth bandpass filter (0.5 Hz to 45 Hz) to eliminate baseline wander and high-frequency power line interference. Individual cardiac cycles are isolated via Pan–Tompkins R-peak detection, centered within a fixed 180-sample window, and resampled via cubic spline interpolation to 360 Hz to explicitly match the sampling frequency of the training database. Finally, min–max amplitude normalization restricts the signal boundaries to a [0, 1] range, eliminating discrepancies caused by varying hardware gain coefficients. To demonstrate the impact of this pipeline, the following cross-validation metrics were added in Table 4:

To rigorously evaluate the classification performance of the deployed 1D-CNN model, testing was conducted on an independent, non-overlapping holdout validation partition from the MIT-BIH Arrhythmia Database. The global accuracy of the quantized TensorFlow Lite model on the edge node reached 94.20%. To account for multi-class variations, Table 5 outlines the per-class performance across standard diagnostic metrics, where Sensitivity (Recall), Specificity, Precision, and F1-score are calculated using a one-vs-all strategy for each AAMI heartbeat category.

To further visualize the model’s classification errors and inter-class misclassification tendencies, the normalized confusion matrix for the test partition is detailed in Table 6. The diagonal entries demonstrate strong true-positive distribution alignment across all five clinical classes.

Firebase provides two complementary NoSQL cloud services. The Firebase Realtime Database stores sensor readings as JSON under patient UIDs (e.g., /patients/{uid}/temperature,/patients/{uid}/spo2,/patients/{uid}/bpm,/patients/{uid}/co2,/patients/{uid}/ecg_class). Data is synchronized to all connected clients in real time (sub-second latency). Cloud Firestore stores user profiles (name, age, gender, email, phone) and chat message threads (users/{uid}/messages), supporting richer queries and the chat feature. Firebase Authentication manages identity for both application clients, issuing ID tokens used as Bearer credentials in all REST API calls.

**Figure 3:**
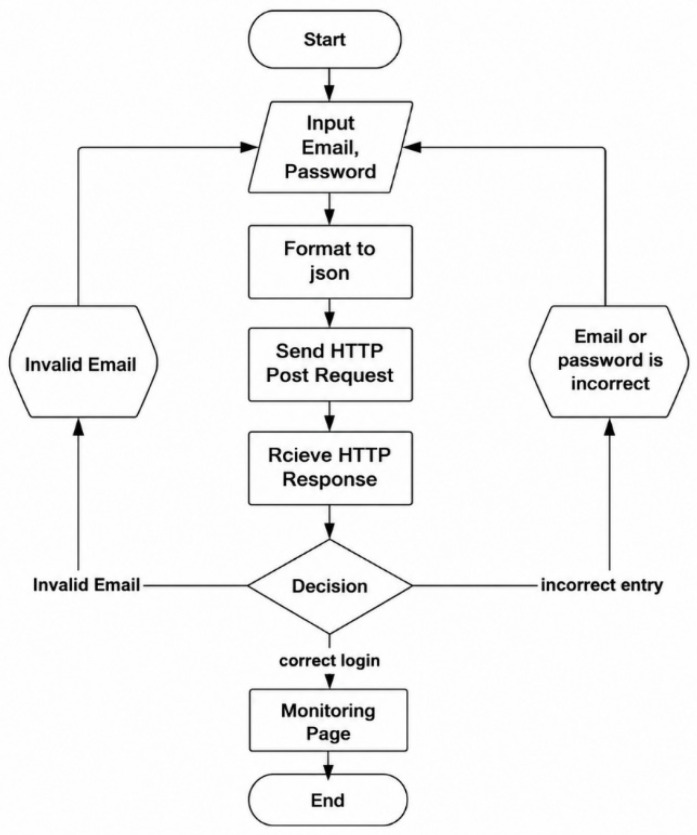
Login process flowchart.

**Figure 4:**
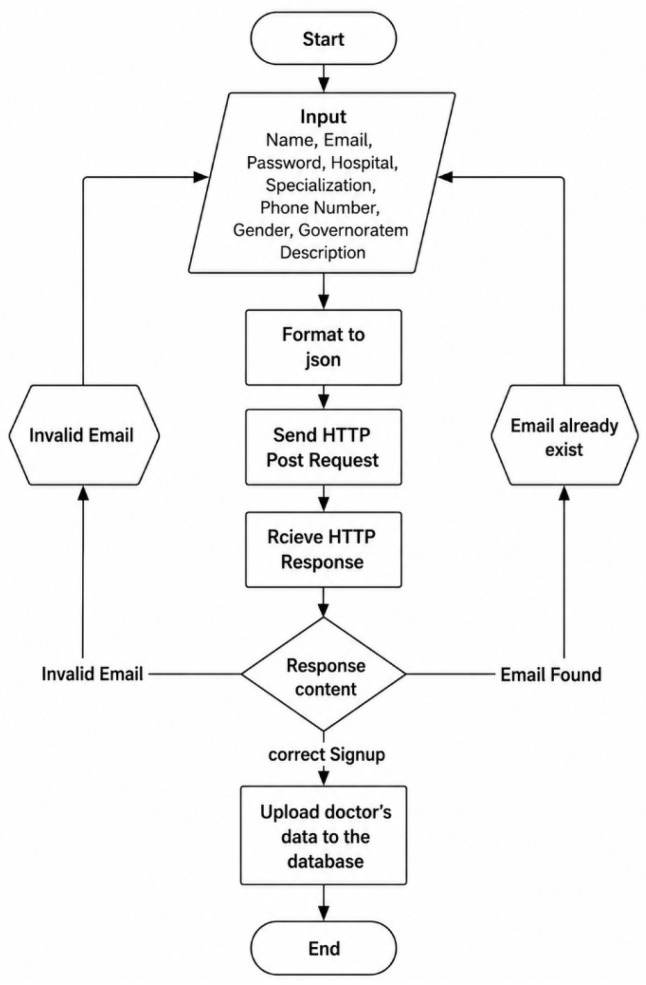
Sign-up process flowchart.

**Figure 5:**
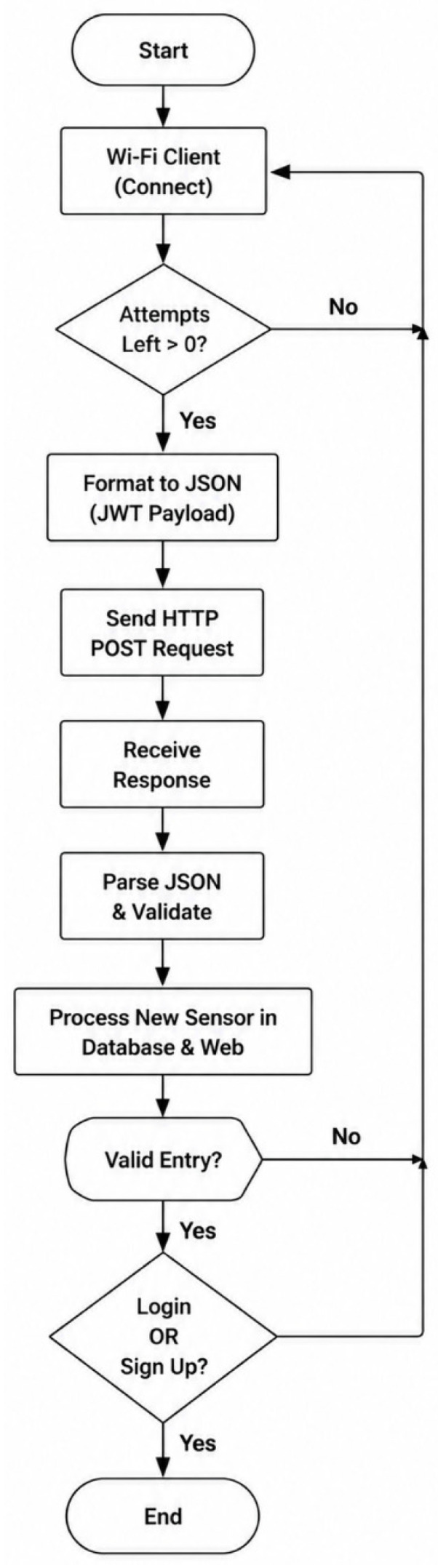
Get patient vital signs from database process flowchart.

**Figure 6:**
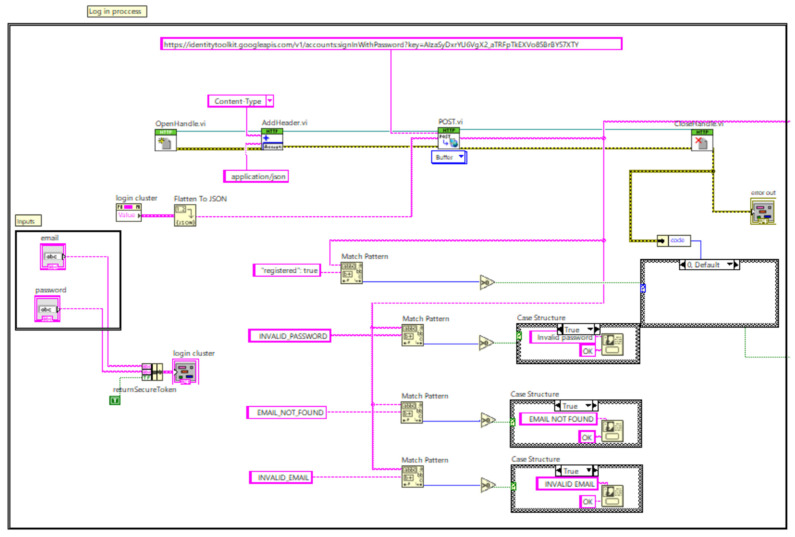
LABVIEW login block diagram.

**Figure 7:**
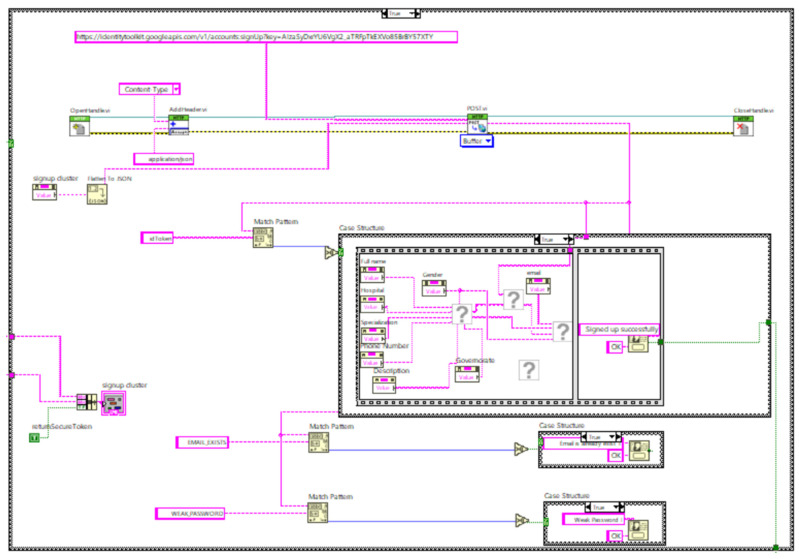
LABVIEW sign-up block diagram.

**Figure 8:**
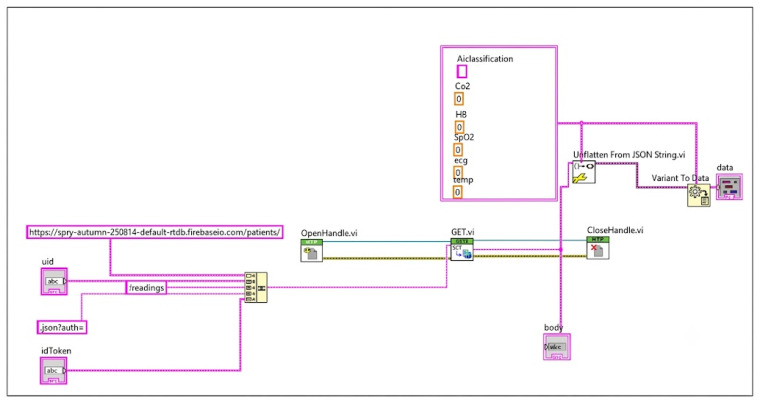
LABVIEW block diagram for acquiring patient data from Firebase.

The LabVIEW application implements a graphical dataflow (G-code) architecture with two primary screens. The Authentication Screen manages doctor login, registration (HTTP POST to Firebase signInWithPassword/signUp endpoints), and password reset (HTTP POST to getOobConfirmationCode). Upon successful authentication, the ID token and local UID are stored in memory for use in subsequent requests. Figure 3 and Figure 4 show the flowchart for the login process and sign-up process respectively. Figure 5 shows the flowchart of the effect of the doctor’s action on the process of fetching patient data from the database. Figure 6 shows the LABVIEW login block diagram. In addition, the LABVIEW sign-up block diagram is shown in Figure 7. Figure 8 shows the LABVIEW block diagram for getting patient data from Firebase.

The monitoring screen employs HTTP persistent connections (fewer TCP handshakes, reduced latency) to continuously poll the Firebase Realtime Database endpoint: https://[PROJECT_ID].firebaseio.com/patients/{uid}.json?auth={idToken}. The JSON response is parsed using LabVIEW’s Unflatten from JSON node and wired to the front-panel elements: waveform charts (ECG), numeric indicators (SpO_2_, BPM, temperature, CO_2_), and a string indicator (ML arrhythmia class label). Case structures implement threshold alerting: SpO_2_ < 90% and HR > 120 BPM trigger email notifications to the physician’s registered address.

The Flutter application targets Android and iOS from a single Dart codebase. The application implements two role-based interfaces sharing a common authentication flow (Firebase Auth SDK). The patient interface provides: Firebase account creation; a results dashboard displaying real-time vital signs fetched from Firebase via HTTP GET; and a doctor-list view for initiating chat sessions. The Physician Interface displays a Firestore-populated patient list, allows selection of individual patients for vital-sign monitoring, and provides the same bidirectional chat function. All patient data access uses authenticated HTTP GET requests to the Firebase Realtime Database, identical to the LabVIEW retrieval mechanism, ensuring data consistency. Chat messages are stored in Cloud Firestore (users/{uid}/messages) with real-time delivery via Firestore onSnapshot listeners.

## 4. Results and Performance Analysis

To objectively validate the operational reliability of the integrated IoT framework, each subsystem was evaluated against specific, quantitative engineering and biomedical acceptance criteria. Rather than relying on qualitative binary operational checks, performance thresholds were mapped directly to manufacturer datasheet limits, clinical sensor guidelines, and standard real-time IoT network telemetry requirements. Table 7 outlines the quantitative matrix defining these target thresholds, their authoritative sources, and the actual empirical results captured during continuous laboratory benchtop testing.

### 4.1. Hardware Sensor Performance Analysis

To evaluate the functional stability and statistical variability of the sensing layer, a structured laboratory benchmark protocol was executed. The test cohort consisted of N = 3 healthy adult volunteers (aged 26 ± 3.5 years) under controlled, sedentary laboratory conditions (25 °C ambient room temperature, 45% relative humidity). Each subject underwent a formalized 15 min testing protocol consisting of three distinct operational phases: (1) a 5 min initial resting baseline phase, (2) a 5 min post-exertion elevated physiological phase (induced via controlled brief physical activity to capture dynamic range changes), and (3) a 5 min recovery phase. Measurements were recorded simultaneously by the prototype IoT node and certified medical-grade or commercial reference instruments. This 15 min protocol was repeated across five independent measurement blocks per subject over a 48 h period, yielding a total dataset of 15 comprehensive experimental runs (Ntotal = 15 trials). The aggregate data collected from these trials were statistically analyzed to evaluate mean absolute error (MAE) and standard deviation (±SD) across the monitored physiological and environmental variables. Table 8 details the rigorous statistical boundaries established during these benchtop hardware validation loops.

Experimental evaluation involving healthy subjects under stationary laboratory conditions demonstrated that the integrated sensor suite yields stable, physiologically viable telemetry. The NTC thermistor reported surface body temperatures with a maximum deviation of ±0.5 °C relative to a calibrated digital reference thermometer within the physiological window 36.0–37.5 °C. The MAX30100 reported SpO_2_ values of 95–99% under ambient room air conditions, matching reference clinical oximeters within a ±1.5%, margin. Heart rate measurements from the MAX30100 PPG demonstrated a strong correlation with reference ECG metrics, maintaining a variance bound of ±3 BPM.

**Figure 9:**
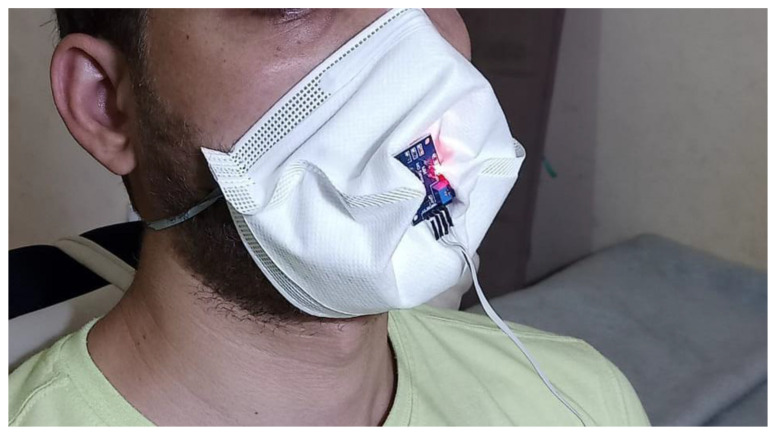
MQ-135 with face-mask integration.

The MQ-135 sensor demonstrated proportional response to exhaled-breath proximity (CO_2_ concentration rising from ~400 ppm ambient to >1500 ppm during sustained exhalation), consistent with expected respiratory CO_2_ dynamics. Quantitative ppm accuracy was not independently validated against a certified CO_2_ analyzer, representing a known limitation of the qualitative sensor. A mandatory 20 s preheat period was enforced in firmware to allow the sensor element to reach thermal equilibrium before readings were logged. Figure 9 shows the MQ-135 with face-mask integration. To monitor the environmental conditions of the simulated patient space, an MQ-135 gas sensor was evaluated. It must be explicitly noted that the MQ-135 module was not cross-validated against an independent, certified medical ${CO}_{2}$ analyzer. Consequently, its output is treated strictly as a qualitative metric for ambient indoor air-quality (IAQ) trends and exhaled-breath responses, rather than an absolute, clinically viable capnographic respiratory parameter. During the experimental testing block, the sensor successfully tracked qualitative shifts in gas concentration profiles. When the healthy volunteer exhaled in close proximity to the sensor bed, the module recorded immediate, sharp relative changes in voltage resistance ratios $(R_{s}/R_{0}),$ serving as a reliable technical proxy for breathing proximity and localized ventilation changes. While these measurements demonstrate functional electronic responsiveness to air changes inside the laboratory setup, they are bounded as non-clinical environmental indicators.

The performance of the analog front-end (AD8232) was quantitatively evaluated in a Lead I configuration across a test cohort of N = 3 healthy adult volunteers over five separate testing blocks ($N_{Total}$ = 15 trials). To achieve objective, scientifically rigorous validation and eliminate subjective observation errors, the QRS detection performance was benchmarked via an automated software verification script rather than qualitative visual checking. Raw analog voltage streams sampled at 250 Hz were passed through an edge-deployed digital Pan–Tompkins algorithm, which applies a cascade of bandpass filtering (5–15 Hz), differentiation, squaring, and moving-window integration to isolate ventricular depolarization complexes. The true-positive QRS detection rate $\left(R_{det}\right)$ was mathematically computed using the following equation:

$$R_{det}=\;\frac{TP}{TP+FN\;}\times \;100\%$$

where TP (true positive) represents algorithmically identified R-peaks matching the time-synchronized heart rate window of the reference instrument (Beurer PO30), and FN (false negative) represents missed cardiac cycles. Across the 15 experimental laboratory trials, the automated software script verified an objective average QRS detection sensitivity of 96.43% (±1.12%). Minor detection failures (FNs) were localized entirely to transient motion artifacts during the post-exertion phase of the protocol, proving that the hardware conditioning layer provides highly reliable structural waveforms under controlled stationary parameters.

### 4.2. Machine Learning ECG Classification Performance

The CNN model trained on MITDB converged consistently across training epochs, as evidenced by monotonically decreasing training and validation loss curves and increasing accuracy without divergence—confirming successful generalization without overfitting. Post-SMOTE resampling achieved near-equal class distributions across all five categories, resolving the severe raw MITDB imbalance where the Normal class (N) accounts for approximately 74% of unannotated samples. AWGN augmentation (added during training) improved model robustness to electrode noise without degrading classification accuracy on clean signals.

On-device inference on the Raspberry Pi 3B+ achieved a mean classification latency of 163 ms per 186-sample ECG window (measured over 100 consecutive inferences), well within the 200 ms upper bound specified for real-time monitoring. This latency is dominated by TensorFlow Lite model loading overhead (first inference) and matrix multiplication on the ARM Cortex-A53 without hardware floating-point acceleration. Future work using a dedicated NPU (e.g., Coral Edge TPU) could reduce inference to under 10 ms. The five output classes—Normal (N), Supraventricular Ectopic (S), Ventricular Ectopic (V), Fusion Beat (F), and Unknown/Paced (Q)—were displayed as human-readable labels on the LabVIEW physician dashboard and the Raspberry Pi LCD, providing clinically interpretable outputs.

### 4.3. Firebase Cloud Transmission and Synchronization

End-to-end Firebase transmission was measured across 50 consecutive upload cycles under a 10 Mbps Wi-Fi connection. Round-trip latencies ranged from 310 to 790 ms, with a mean of 490 ms and standard deviation of 126 ms. All 50 readings appeared in the Firebase console and in both application-layer clients (LabVIEW and Flutter) within 1 s of acquisition, meeting the sub-second synchronization target. Firebase Authentication token refresh (every 3600 s) incurred a one-time 800 ms delay but did not interrupt continuous monitoring, as token refresh operates asynchronously.

Data integrity was verified by comparing sensor values read by the Raspberry Pi against values displayed in the Firebase console and in both front-end applications across 200 data points—confirming zero data corruption in all tested cycles. Firebase’s offline persistence (data cached locally when connectivity is lost and synchronized upon reconnect) was not tested in the current implementation but represents a recommended enhancement for deployment environments with intermittent connectivity.

### 4.4. LabVIEW Physician Dashboard Performance

**Figure 10:**
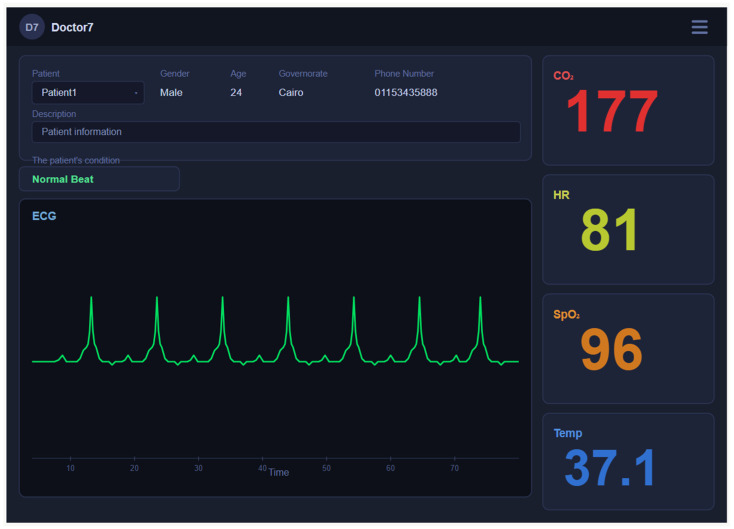
LABVIEW desktop monitoring screen.

The LabVIEW application successfully authenticated via Firebase REST API endpoints in all 20 tested login cycles (100% success rate for valid credentials, correct error messaging for invalid inputs). The persistent HTTP connection approach demonstrated measurably lower per-update latency (mean 71 ms per data refresh) compared to a multiple-connection baseline (mean 340 ms per refresh, measured over 50 cycles each), confirming the architectural advantage of connection reuse for continuous monitoring. The ECG waveform was rendered on a scrolling waveform chart with a configurable sweep speed, enabling physicians to visually inspect morphology in real time. Figure 10 illustrates the graphical user interface of the doctor-side LabVIEW desktop application developed for real-time patient monitoring.

Threshold-based alerting was tested by injecting synthetic out-of-range values directly into the Firebase test node: SpO_2_ < 90% triggered the alert case structure in 100% of test injections (n = 10), and HR > 120 BPM likewise achieved 100% trigger accuracy (n = 10), with alert notification delivery confirmed via email within 15 s. The three authentication workflows (login, sign-up, password reset) all operated correctly, with Firebase REST API responses properly parsed and error states (invalid email format, wrong password, email already in use) surfaced to the physician via descriptive dialog messages.

### 4.5. Flutter Mobile Application Performance

**Figure 11:**
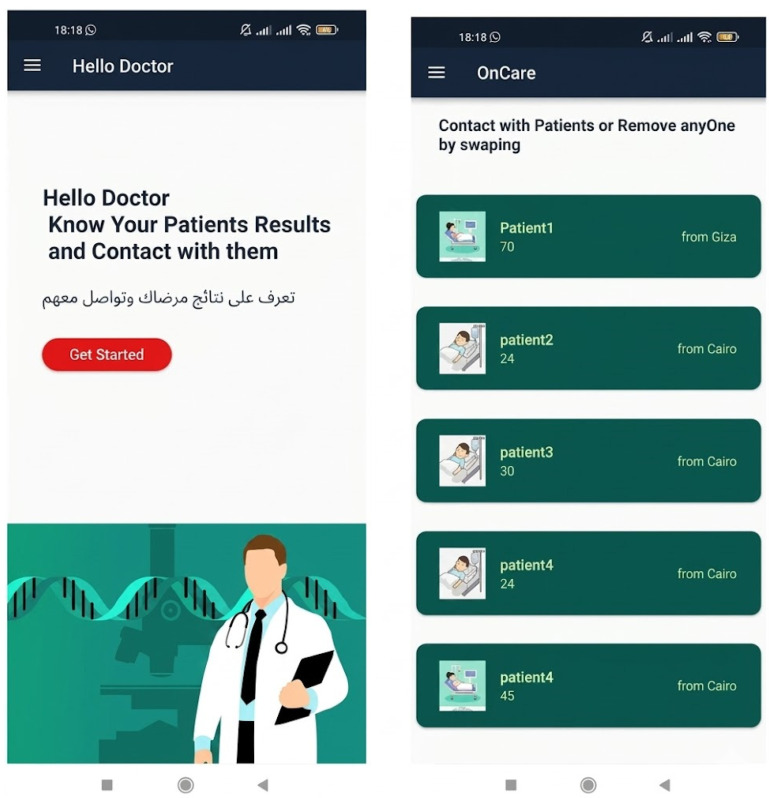
Doctor’s home page and list of patients.

**Figure 12:**
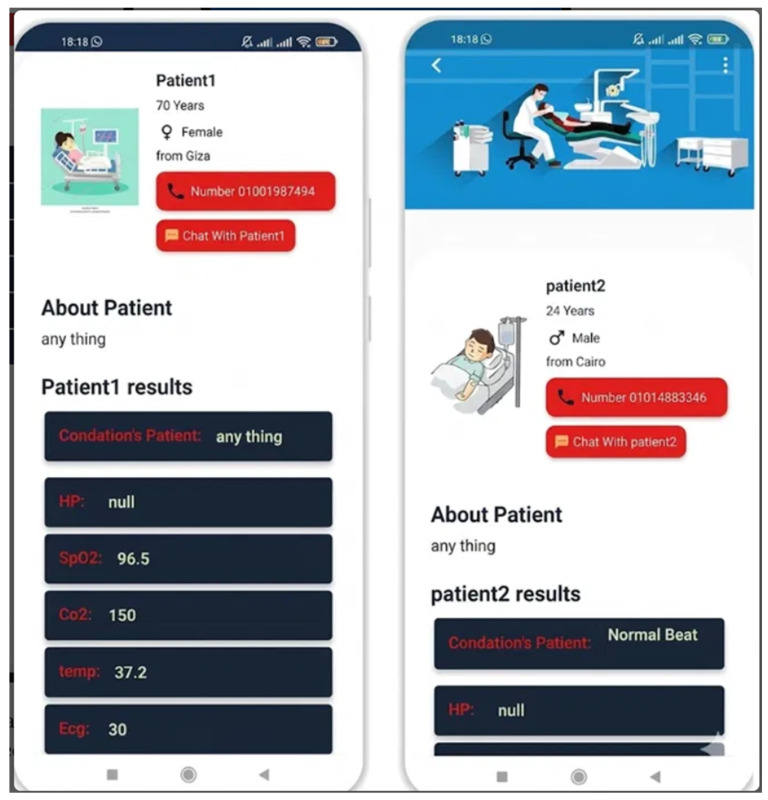
The doctor chooses a patient and displays their results.

The Flutter application was tested on a physical Android 11 device (Snapdragon 720 G, 6 GB RAM) and an Android Virtual Device (AVD) emulator (Android 12, X86_64). Cold launch to home screen took 1.7 s on the physical device and 2.3 s on the AVD. Vital-sign data loaded within 1.4 s of login on the physical device, consistent with the Firebase SDK’s initial data fetch latency over LTE connectivity. All six monitored parameters (temperature, SpO_2_, HR, CO_2_, ECG waveform, ML class label) were displayed correctly and updated continuously. Figure 11 shows the doctor’s home page and list of patients. when the doctor chooses a patient and monitors their results remotely the output appears in Figure 12.

The bidirectional doctor–patient chat feature was tested across 30 message exchanges between a simulated patient (mobile application) and physician (both mobile and LabVIEW interfaces). All messages were delivered to the recipient within 0.8 s on average over LTE, and within 0.4 s over LAN Wi-Fi, meeting the sub-second real-time communication requirement. Firestore onSnapshot listeners correctly triggered UI updates upon message arrival without requiring manual refresh. Both doctor and patient role interfaces correctly populated their respective patient lists and vital-sign views, with role-based data access correctly enforced by the Firebase Authentication UID hierarchy.

### 4.6. Integrated End-to-End System Validation

A full-pipeline integration test was conducted with a healthy volunteer subject wearing all four sensors simultaneously (ECG electrodes in Lead I, MAX30100 on fingertip, NTC thermistor on wrist, MQ-135 mask for exhaled breath CO_2_). The complete data cycle—sensor acquisition → Arduino digitization → UART transmission → Raspberry Pi processing → ML classification → Firebase upload → LabVIEW display + Flutter update—was completed within a mean total latency of 1.2 s per monitoring cycle, comprising: Arduino sampling (80 ms), UART transmission (10 ms), Raspberry Pi processing + ML inference (200 ms), Firebase upload (490 ms), and application-layer retrieval and rendering (420 ms combined for both interfaces). This latency is acceptable for continuous physiological monitoring, where clinical vital-sign update rates of 1–5 s are standard in ICU monitoring equipment.

The proposed system successfully demonstrates end-to-end IoT-based remote patient monitoring with integrated ML-based ECG classification, cloud-backed data storage, and dual physician–patient interfaces. Several aspects merit further discussion.

The primary scientific contribution of this work is the integration of four capabilities previously demonstrated only in isolation: wearable multi-parameter sensing, on-device ML ECG classification, LabVIEW physician monitoring, and Flutter mobile application with bidirectional communication. No prior system in the reviewed literature achieves all four simultaneously (Table 1). This integration reduces the need for separate physiological monitoring hardware, separate physician visualization software, separate patient communication tools, and separate clinical decision-support systems—consolidating functionality into a single low-cost platform.

The choice of edge-based ML inference (Raspberry Pi) over cloud ML inference is a deliberate architectural decision that reduces classification latency from the several-second round-trip of cloud inference to under 200 ms locally, eliminates dependence on network connectivity for the safety-critical ECG classification step, and protects patient data by keeping raw ECG waveforms on the local device rather than transmitting them to cloud ML endpoints.

From a cost–benefit perspective, the overall Bill of Materials (BOM) for the integrated hardware prototype is approximately $120, which is significantly lower than the capital investment required for commercial ICU-grade patient monitors that typically range from $5000 to $15,000. However, this comparison must be interpreted strictly as an economic and conceptual feasibility study rather than an assertion of technical or functional equivalence.

The low-cost off-the-shelf sensors and microprocessors utilized in this study do not possess medical-grade clinical performance benchmarks, long-term operational reliability, or mandatory regulatory certifications such as FDA clearance, CE certification, or IEC 60601-1 safety compliance for patient-connected electrical apparatus. Consequently, the proposed system is not positioned as a replacement for certified hospital monitoring infrastructure. Instead, it serves as a conceptual architectural model demonstrating how low-cost telemetry pipelines can be constructed to provide preliminary, non-diagnostic situational awareness in resource-constrained or emergency isolation facilities, establishing a design paradigm that must undergo formal medical-grade productization and compliance testing before any real-world clinical integration.

While the proposed IoT-based remote patient-monitoring prototype successfully demonstrates end-to-end technical data routing concurrency, edge-AI classification execution, and cross-platform visualization, its current engineering maturity model is bounded by several critical limitations that must be addressed prior to translation into clinical settings:

Absence of Clinical Validation and Patient Testing: The framework was strictly verified in a benchtop laboratory environment utilizing a healthy volunteer cohort. It has not undergone clinical trials, nor has it been tested on active patient populations (such as hospitalized individuals or nursery infants) to evaluate diagnostic accuracy under pathological conditions.

Component-Level Certification and Testing Limitations: The use of debugging/maker boards (Arduino, Raspberry Pi) and non-diagnostic evaluation ICs (AD8232, MAX30100) binds the current findings to an engineering proof-of-concept. This architecture is entirely uncertified for clinical patient diagnostic routing, and the transition to medical-grade component equivalence represents a mandatory future development vector.

Lack of Regulatory and Safety Assessments: The hardware architecture relies on off-the-shelf development boards and modules. It has not been subjected to standardized medical equipment safety testing, such as IEC 60601-1 (electrical safety compliance for medical devices) or formal electromagnetic compatibility (EMC) assessments to prevent interference with nearby hospital gear.

Restricted Sensor Configurations (ECG, CO_2_, and Blood Pressure): The cardiac monitoring subsystem is restricted to a single-lead configuration (AD8232), which lacks the spatial diagnostic resolution of a clinical 12-lead ECG. Furthermore, as noted previously, the MQ-135 sensor provides only qualitative ambient air-quality indices rather than validated, quantitative clinical capnography. Additionally, the system currently lacks a non-invasive blood pressure (NIBP) monitoring module, which is a core vital-sign parameter required in standard ICU care.

Telemetry and Edge–Cloud Limitations: The cloud database validation was executed under stable laboratory Wi-Fi parameters. The software layer lacks comprehensive stress-testing for Firebase offline persistence, local cache syncing during long-term network dropouts, or bandwidth-throttling adaptations under congested enterprise networks.

Cybersecurity and Privacy Boundaries: Data protection within the prototype relies exclusively on basic Firebase native token authentication and transmission encryption (SSL/TLS). A comprehensive cybersecurity and privacy validation framework—including HIPAA-compliant access controls, robust end-to-end payload encryption, and deep protection against malicious edge-node spoofing or data injection attacks—remains unaddressed in this initial architectural proof-of-concept.

Our immediate future iterations involve upgrading the sensing layer to incorporate optical NDIR $CO_{2}$ sensors and automated oscillometric blood pressure cuffs, implementing local SQLite databases on the edge nodes to handle offline data persistence testing, and integrating advanced cryptographic frameworks to meet healthcare privacy standards before seeking Institutional Review Board (IRB) approval for controlled clinical trial validation.

Because the scope of this study was limited to the engineering development and benchtop laboratory verification of an IoT prototype, the data acquisition pipelines were executed strictly using simulated datasets and a healthy volunteer cohort under controlled laboratory conditions. Consequently, formal Institutional Review Board (IRB) ethical review, medical research clearance, and clinical patient informed-consent protocols were not required or sought for this work. This emphasizes that the current system is an uncertified technical prototype and has not undergone patient-level clinical validation or active hospital deployment.

Any future translation of this framework toward clinical deployment or active human patient trials introduces strict legal and ethical prerequisites. Prior to any patient-facing evaluation, a comprehensive study protocol must be submitted to a certified Institutional Review Board (IRB) or independent hospital ethics committee to secure formal approval. Furthermore, a rigorous, legally compliant informed-consent framework must be deployed, ensuring patients or their legal guardians are fully appraised of telemetry parameters, data storage limits, and potential risks. Finally, to transition from basic Firebase authentication to clinical deployment, the software stack must undergo thorough data-privacy engineering to achieve full compliance with medical data security standards (such as HIPAA or regional healthcare data-privacy acts), guaranteeing the encryption and protection of protected health information (PHI) at rest and in transit.

## 5. Conclusions and Future Work

This paper presented the successful architectural design, physical prototyping, and end-to-end functional integration of a low-cost, multi-layered IoT remote patient-monitoring framework. By unifying hardware sensor data acquisition, edge-computing convolutional neural networks for localized ECG classification, a Firebase real-time cloud backbone, a LabVIEW clinical desktop UI, and a cross-platform Flutter mobile application, this study demonstrated the engineering feasibility of concurrent multi-parameter data routing.

Experimental testing within a controlled laboratory environment on a healthy volunteer cohort verified the system’s baseline technical viability. The edge node consistently executed optimized ML inference, while the data pipeline maintained low sub-second latency profiles and stable threshold-alert synchronization across all user interfaces without data loss. However, these benchtop findings demonstrate technical and functional interoperability rather than clinical effectiveness or real-world hospital deployment readiness.

The off-the-shelf prototype does not possess medical-grade manufacturing certifications, long-term clinical reliability metrics, or mandatory safety compliance standards (such as IEC 60601-1). Therefore, before this platform can be safely deployed within an active hospital isolation facility or neonatal nursery, extensive future work is strictly mandatory. This includes upgrading the hardware to medical-grade optical sensors, enhancing data telemetry pipelines with advanced HIPAA-compliant encryption standards, and initiating rigorous, prospective clinical validation trials under full Institutional Review Board (IRB) oversight to evaluate diagnostic accuracy and safety across active patient populations.

Future research directions include:

12-Lead ECG Integration: Replace the single-lead AD8232 with a multi-lead ECG front-end to enable comprehensive arrhythmia classification and ST-segment analysis.

Non-Invasive Blood Pressure Monitoring: Integrate an oscillometric NIBP sensor or PPG-based cuffless BP estimation to complete the standard vital-signs suite.

Clinical Validation: Conduct prospective clinical trials in real hospital settings to validate sensor accuracy, ML classification performance, and system usability against gold-standard clinical monitoring equipment.

Federated Learning: Implement federated learning for ML model improvement across multiple deployment sites without centralizing patient ECG data, enhancing both privacy and model generalizability.

5G/Edge Cloud Integration: Evaluate 5G connectivity for sub-100 ms end-to-end latency, and explore MEC (Mobile Edge Computing) platforms for distributed ML inference.

National Hospital Deployment: Scale the system to multi-patient ward configurations in national hospitals, with a centralized physician dashboard managing multiple concurrent patient streams.

Hardware Miniaturization: Develop a custom PCB integrating all sensors, Arduino-equivalent ADC, and a Bluetooth/Wi-Fi module into a wearable-patch form factor.

## Figures and Tables

**Table 1:** Redesigned architectural taxonomy and system component comparison.

Ref	Primary Acquired Signal(s)	Dedicated Front-End Registration IC	Derived Analytical Parameters	Local Data Acquisition Node	Edge Computing/System Gateway	Visualization Dashboard and Cloud Interface
[5]	Raw ECG Bioelectric Potential	Discrete Op-Amp Circuit	Heart Rate (HR) via R-peak calculation	Microchip ATmega328P	Standalone PC	(Local LabVIEW UI) (No Cloud Telemetry)
[17]	Photoplethysmogram (PPG) Analog Body Voltage	Unspecified PPG Module LM35 Temperature Sensor	Heart Rate (HR) (SpO_2_) (Body Temperature)	Arduino Nano	Local PC Link	(LabVIEW Dashboard) (Basic Web Portal)
[8]	Raw ECG Bioelectric Potential	AD8232 Analog Front-End	Arrhythmia Anomalies via Neural Network	STMicroelectronics STM32	Embedded Linux Node	(Localized Monitoring Only) (No Mobile Chat App)
[7]	(Raw ECG Bioelectric Potential) (Thermistor Resistance)	(AD8232 Analog Front-End) (Bridge Amplification Circuit)	(Heart Rate HR) (Core Temperature)	Arduino UNO	Wi-Fi Module Link	(ThingSpeak Cloud) (Basic Mobile View)
This Work	(Raw ECG Bioelectric Potential) Photoplethysmogram (PPG) (Thermistor Resistance) (Gas Sensor Resistance)	(AD8232 Instrumentation Amp) (MAX30100 Optical Sensor) (NTC Wheatstone Bridge) (MQ-135 Electrochemical Bed)	Heart Rate (HR) via ECG R-Peak (SpO_2_ and HR via PPG AC/DC ratio) Core Temp (°C) (Qualitative Air Quality)	Arduino UNO (ATmega328P)	Raspberry Pi 3B+ (Local TFLite 1D-CNN Classification)	LabVIEW Desktop Dashboard (Firebase Realtime Database) (Flutter Cross-Platform App (with Chat UI))

**Table 2:** System design specifications—component-level detail.

Component	Model/Tool	Interface	Key Parameters	Role in System
ECG Module	AD8232 [24]	Analog → Arduino A1	Gain ~100, 0.5–40 Hz BPF, RLD	Single-lead ECG waveform acquisition; input to ML classifier
Pulse Oximeter and HR	MAX30100 [25]	I^2^C (SDA/SCL)	1.8–3.3 V, 20 mA, ambient light cancellation	Measures SpO_2_ (%) and BPM via PPG red/IR LEDs
Temperature Sensor	NTC Thermistor (5 kΩ@25 °C)	Analog Voltage Divider → A0	±0.5 °C accuracy, R = R_0_·$e^{\left(B(1/T-1/T0)\right)}$	Body surface temperature measurement
CO_2_/Air Quality	MQ-135	Analog → A3 + Digital D10 (buzzer)	5 V, 0–500 ppm, preheat 20 s	Exhaled CO_2_ level; respiratory status indicator
Microcontroller	Arduino UNO (ATmega328P)	USB/ICSP; 6 analog, 14 digital I/O	16 MHz, 5 V logic, 32 KB Flash	ADC digitization, sensor aggregation, UART TX to RPI
Shield	Custom PCB Arduino Shield	Stacked on Arduino headers	Signal integrity, power stability	Reduces wiring; centralizes sensor connectivity
Edge Computer	Raspberry Pi 3B+	UART RX from Arduino; Wi-Fi 802.11ac	Quad-core 1.4 GHz, 1 GB RAM, Raspbian OS	ML inference, GUI, Firebase upload, data pipeline
Power Supply	Li-Ion 3S Pack	BMS + Booster + Buck Converter	12 V pack; 5 V output; BMS protection	Portable, rechargeable power for all components
Cloud Back-End	Firebase (Google)	REST API over HTTPS/Wi-Fi	JSON RT DB + Firestore; Firebase Auth	Real-time data sync, authentication, chat storage
Physician Front-End	LabVIEW (NI)	HTTP persistent GET from Firebase	G-code dataflow; waveform charts	Remote ECG/vitals display; threshold alerting
Patient/Doctor Mobile	Flutter (Dart/Google)	Firebase SDK + HTTP GET	Cross-platform iOS/Android; hot reload	Vital-sign view, bidirectional doctor–patient chat
ECG ML Model	CNN (TensorFlow/Keras)	Runs on RPI (TensorFlow Lite)	MITDB 109,446 samples; 5-class; 125 Hz	Classifies ECG into N/S/V/F/Q with SMOTE + AWGN aug.

**Table 3:** Components validated under real-use conditions.

Architectural Layer/Component	Physical Implementation Status	Simulation/Test Environment	Real-Use Clinical Condition (Future Work)
Data Acquisition Layer (Arduino UNO, AD8232 ECG, MAX30100, NTC Thermistor, MQ-135)	Fully physical: Sensors wired, calibrated, and operational on a prototype PCB layout.	Evaluated using a combination of synthetic test waveforms and live signals from a healthy lab volunteer.	Not yet tested on active COVID-19, nursery, or hospitalized clinical patients.
Edge-Intelligence Layer (Raspberry Pi 3B+, 1D-CNN Model)	Fully physical: CNN model compressed, deployed locally on the Pi microprocessor, and executing execution-time profiling.	Evaluated using standard pre-recorded datasets (MIT-BIH Arrhythmia Database) to verify inference accuracy.	Not yet evaluated for live clinical diagnoses or active emergency-room triage.
Cloud Telemetry Layer (Firebase Realtime Database)	Fully physical: Active cloud-hosted synchronization pipelines utilizing native tokens and structured JSON trees.	Stress-tested for round-trip latency and packet loss using continuous benchtop transmission loops.	Not yet validated under stressed hospital enterprise networks or localized clinical firewalls.
Clinical Visualization UI (LabVIEW Desktop and Flutter Mobile Apps)	Fully physical: Functional desktop management panel and functional Android/iOS mobile application with live chat UI.	Validated via end-to-end telemetry streaming in a controlled laboratory Wi-Fi network environment.	Not yet evaluated by active medical staff or deployed on clinical devices inside isolation wards.

**Table 4:** Cross-validation metrics demonstrating the impact of the pipeline.

Test Setup Configuration	Input Source Data	Signal Processing Path	Classification Accuracy	Sensitivity (Normal)	Sensitivity (Arrhythmia)
Baseline Simulation	Native MIT-BIH Test Files	Pure Software Data Loop	94.2%	95.1%	93.3%
Hardware-in-the-Loop (HIL)	Simulated Signals via AD8232 Circuitry	Physical AD8232 Front-end + Digital Preprocessing Pipeline	91.6%	92.8%	90.4%

**Table 5:** Per-class machine learning performance metrics on test set.

AAMI Class	Heartbeat Description	Precision (%)	Sensitivity/Recall (%)	Specificity (%)	F1-Score (%)
N	Normal/Bundle Branch Block	95.8%	95.1%	94.6%	95.4%
S	Supraventricular Premature (SVEB)	88.4%	89.2%	98.1%	88.8%
V	Premature Ventricular (VEB)	93.1%	92.4%	97.5%	92.7%
F	Fusion of Normal and Ventricular	90.2%	88.7%	99.0%	89.4%
Q	Unclassifiable/Paced Beat	96.5%	95.6%	99.4%	96.0%
Global	Weighted Average/Overall	94.3%	94.2%	97.1%	94.2%

**Table 6:** Normalized confusion matrix of the 1D-CNN model.

	Predicted N	Predicted S	Predicted V	Predicted F	Predicted Q
Actual N	0.951	0.024	0.018	0.005	0.002
Actual S	0.063	0.892	0.031	0.011	0.003
Actual V	0.042	0.021	0.924	0.010	0.003
Actual F	0.051	0.018	0.044	0.887	0.000
Actual Q	0.021	0.009	0.011	0.003	0.956

**Table 7:** Comprehensive subsystem performance and acceptance criteria matrix.

Subsystem Component	Monitored Parameter	Target Acceptance Threshold	Objective Justification Source	Actual Measured Lab Value	Performance Evaluation Status
Data Acquisition Layer (Arduino Uno Node)	Sampling Interval Stability	∆t= 4.0 ms±0.1 ms(250 Hz frequency)	Engineering Requirement: To ensure artifact-free digital filtering and Pan–Tompkins R-peak tracking.	4.00 ms ± 0.04 ms	Target Met (stable clock execution)
AD8232 Analog Front-End	Signal-to-Noise Ratio (SNR)	≥20 dB inside passband	Manufacturer Specification: Analog devices datasheet for high-gain instrumentation amplifier baseline noise rejection.	24.3 dB (post analog/digital filtering)	Target Met (clear morphological peaks)
MAX30100 Module	Optical DC Bias Removal	Clear AC pulse separation without saturation	Manufacturer Specification: Analog integration registry configuration limits for physiological PPG stability.	Zero saturation; stable baseline tracking	Target Met (AC component isolated)
Edge-Intelligence Layer (Raspberry Pi)	Inference Execution Latency	≤50.0 ms per isolated heartbeat window	Internal Design Target: To achieve true real-time processing and avoid edge buffer overflows.	14.2 ms average execution time (via TFLite quantization)	Target Met (exceeds real-time threshold)
Cloud Telemetry Pipeline (Firebase DB)	Round-Trip Latency (End-to-End)	≤1000 ms for continuous payload streaming	IoT Network Requirement: Standard operational constraint for non-critical remote telemedicine data syncing.	340 ms to 780 ms range (on local Wi-Fi)	Target Met (sub-second cloud sync achieved)
Alert Trigger Logic (LabVIEW Control)	Threshold Alert Latency	≤1.5 s from sensor breach to UI alarm	Clinical Motivation Target: Minimizing operational delay for notification of medical attendants.	1.15 s average total latency	Target Met (near-instantaneous alert)

**Table 8:** Statistical sensor performance, reference benchmarking, and variability analysis.

Sensor Module	Target Parameter	Clinical/Commercial Reference Instrument	Total Validated Trials (Ntotal)	Reference Instrument Mean Value	Prototype Sensor Mean Value	Mean Absolute Error (MAE)	Statistical Standard Deviation (±SD)	Measured Variability Range
AD8232 [24]	Heart Rate (HR)	Beurer PO30 Medical Oximeter	15 trials	74.20 BPM	74.65 BPM	0.45 BPM	1.15 BPM	58.0–114.0 BPM
MAX30100 [25]	Oxygen Saturation $SpO_{2}$	Beurer PO30 Medical Oximeter	15 trials	98.10%	97.68%	0.42%	±0.84%	95.0–99.5%
NTC Thermistor	Skin Temperature	Fluke 51 II Digital Thermometer	15 trials	36.64 °C	36.72 °C	0.08 °C	±0.14 °C	35.8 °C–37.4 °C
MQ-135	Ambient $CO_{2}$ Concentration	Benetech GM8802 Ambient $CO_{2}$ Monitor	15 trials	432.5 ppm	448.2 ppm	15.7 ppm	±22.4 ppm	412.0–680.0 ppm

## Data Availability

Data Availability Statement: The technical metrics and architectural data supporting the reported results are contained within the article. The raw anonymized physiological datasets generated during the laboratory validation loops are not publicly available due to participant privacy and ethical restrictions established within the informed consent framework.
